# A Book of Detachable Diet Lists and a Sick-room Dietary

**Published:** 1896-01

**Authors:** 


A BOOK OF DETACHABLE DIET LISTS AND A SICK-ROOM DIETARY. Compiled by Jerome B. Thomas, A. B., M. D. Price, $1.50. Published by W. B. Saunders, 925 Walnut street, Philadelphia. 1895.

This book of detachable leaves, each one containing a list of articles of diet, and ten separate lists to choose from, will be found a great convenience to the physician, and it is really a time saver. There are ten different kinds of lists, from which may be selected one that, with perhaps a little alteration, wil l suit almost any case the doctor meets.

Each list is arranged with reference to a certain class of diseases, as follows: List 1, albuminuria; 2, anæmia; 3, constipation; 4, diabetes; 5, diarrhœa; 6, dyspepsia; 7, fevers; 8, gout or uric acid diathesis; 9, obesity; 10, tuberculosis.

It is not expected that these lists will suit all cases, in fact, it may be necessary to make some alterations in almost all cases, but the plan is for the physician to detach one leaf from the list nearest suited to the patient he has under treatment, check off such articles as he thinks unsuited to this particular case, make such additions as he wishes, and hand the corrected list to the nurse. All this can be done in a few seconds, or minutes at most, when, without the printed list, he would have to spend ten times the amount of time writing out a suitable dietary, or run the risk of having his patient improperly fed. At the end of the volume are a number of leaves giving full instructions for preparing the various articles of diet most suited to the sick. These leaves can also be detached, and one given to the nurse. H.

